# The Impact of Protein Phosphorylation on Chlamydial Physiology

**DOI:** 10.3389/fcimb.2016.00197

**Published:** 2016-12-22

**Authors:** Ja E. Claywell, Lea M. Matschke, Derek J. Fisher

**Affiliations:** Department of Microbiology, Southern Illinois UniversityCarbondale, IL, USA

**Keywords:** *Chlamydia*, reversible phosphorylation, kinase, phosphatase, phosphoprotein, signaling

## Abstract

*Chlamydia* are Gram negative bacterial pathogens responsible for disease in humans and economically important domesticated animals. As obligate intracellular bacteria, they must gain entry into a host cell where they propagate within a parasitophorous organelle that serves as an interactive interface between the bacterium and the host. Nutrient acquisition, growth, and evasion of host defense mechanisms occur from this location. In addition to these cellular and bacterial dynamics, *Chlamydia* differentiate between two morphologically distinct forms, the elementary body and reticulate body, that are optimized for either extracellular or intracellular survival, respectively. The mechanisms regulating and mediating these diverse physiological events remain largely unknown. Reversible phosphorylation, including classical two-component signaling systems, partner switching mechanisms, and the more recently appreciated bacterial Ser/Thr/Tyr kinases and phosphatases, has gained increasing attention for its role in regulating important physiological processes in bacteria including metabolism, development, and virulence. Phosphorylation modulates these events via rapid and reversible modification of protein substrates leading to changes in enzyme activity, protein oligomerization, cell signaling, and protein localization. The characterization of several conserved chlamydial protein kinases and phosphatases along with phosphoproteome analysis suggest that *Chlamydia* are capable of global and growth stage-specific protein phosphorylation. This mini review will highlight the current knowledge of protein phosphorylation in *Chlamydia* and its potential role in chlamydial physiology and, consequently, virulence. Comparisons with other minimal genome intracellular bacterial pathogens also will be addressed with the aim of illustrating the importance of this understudied regulatory mechanism on pathogenesis and the principle questions that remain unanswered.

## Introduction

*Chlamydia* are obligate intracellular bacteria that are responsible for diseases in humans and animals creating a significant burden on global health and national economies (Horn, 2008; World Health Organization, 2016a,b). These pathogens undergo a biphasic developmental cycle, transitioning between two morphologically and functionally distinct forms known as the infectious elementary body (EB) and the replicative reticulate body (RB) (Abdelrahman and Belland, 2005). Infection begins with EB attachment to a mucosal epithelial cell. Upon contact, the type 3 secretion system (T3SS) secretes prepackaged effector proteins to induce EB internalization into a parasitophorous organelle termed the inclusion (Moore and Ouellette, 2014). Following entry, *Chlamydia* exploits host cell trafficking machinery to exit the endocytic pathway and migrate to a perinuclear position where the inclusion can interact with the exocytic pathway (Hackstadt, 2000). The EBs differentiate into metabolically active RBs that grow and divide via a polarized budding-like process (Abdelrahman et al., 2016). Both bacterial and host proteins are incorporated into the growing inclusion, and numerous bacterial proteins are secreted into the inclusion lumen and host cell cytoplasm leading to host protein recruitment, nutrient acquisition, maintenance of anti-apoptotic pathways, and modulation of innate immune mechanisms (Bastidas et al., 2013). RBs eventually differentiate back into EBs and exit the host cell via lysis or inclusion extrusion approximately 48–72 h post-infection (Hybiske and Stephens, 2007). Exposure to stress, including antibiotics, IFNγ, or iron deprivation during intracellular growth, can induce a reversible persistent state characterized by formation of viable, non-dividing, aberrant RBs (Wyrick, 2010). Persistent *Chlamydia* undergo differential gene expression depending on the persistence-inducing stimuli (Belland et al., 2003a; Goellner et al., 2006; Mäurer et al., 2007).

Despite the essentiality of development to infection, making it an ideal target for therapeutics, relatively little is known about the signals and mechanisms regulating this process. *Chlamydia* possess three sigma factors, σ^66^, σ^28^, and σ^54^ (encoded by *rpo*D, *rps*D, and *rpo*N, respectively), and exhibit developmental stage-specific gene expression patterns corresponding to an early, middle, and late stage of infection (Stephens et al., 1998; Shaw et al., 2000; Belland et al., 2003b). We hypothesize that post-translational regulatory mechanisms help regulate and mediate chlamydial development in addition to the more classical transcriptional regulators that have been described for *Chlamydia*.

**Figure 1:**
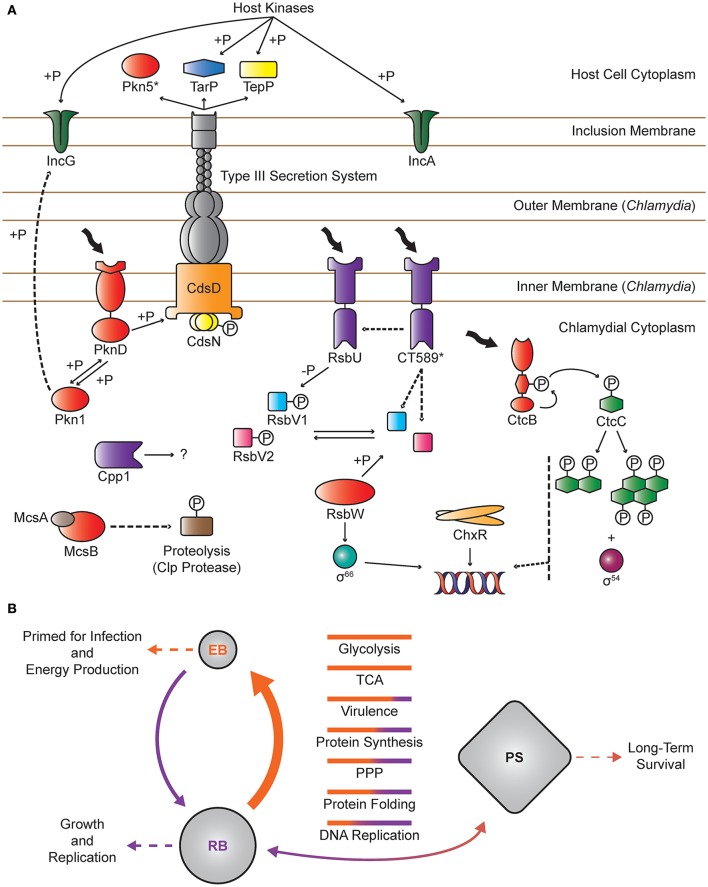
**Overview of Protein Phosphorylation in ***Chlamydia***. (A)** Individual proteins: *Chlamydia* spp. encode two validated Hank's type kinases, Pkn1 and PknD. Pkn1 is predicted to reside in the cytoplasm and may interact with IncG, an inclusion membrane protein. PknD is an integral membrane protein that most likely binds to an unidentified ligand and interacts with CdsD, a component of the T3SS apparatus. CdsN, the T3SS ATPase, may also be modified by phosphorylation. Both Pkn1 and PknD have been shown to undergo autophosphorylation and interact with each other. Pkn5 is predicted to be a pseudokinase that is secreted via the T3SS. *Chlamydia* also encode three protein Ser/Thr phosphatases. Cpp1 is a broad specificity PP2C that may function intracellularly in concert with Pkn1 and PknD. The chlamydial PSM consists of two sensor phosphatases (RsbU and CT_589), two anti-anti-sigma factors (RsbV1 and RsbV2), an anti-sigma factor (RsbW), and σ^66^. CT_589 is a paralog of RsbU that lacks a functional phosphatase domain and is proposed to interact with RsbU and/or to sequester RsbV1/2. CtcB and CtcC comprise the only complete TCSS in *Chlamydia* spp. The HK, CtcB, undergoes autophosphorylation on a conserved histidine residue, which is transferred to the RR, CtcC. While CtcC lacks a DNA binding domain, it is able to undergo oligomerization in the absence of DNA, which is then thought to interact with σ^54^ to promote transcription. ChxR is an atypical response regulator, lacking a cognate HK and the conserved phospho-receiving aspartate residue. ChxR undergoes dimerization, binds DNA, and likely serves as a transcriptional activator. McsB, upon activation by McsA, mediates protein homeostasis by phosphorylating proteins and targeting them for degradation by the Clp protease. TarP, TepP, IncA, and IncG are T3SS effector proteins that are phosphorylated by host kinases and are involved in cell invasion and inclusion development. Protein kinases and the pseudokinase Pkn5 are colored in red. Proteins with phosphatase activity or phosphatase-like domains (CT_589) are colored in purple. Known interactions are represented by solid lines and hypothetical interactions are shown by dashed lines. **(B)** Global patterns: The EB phosphoproteome is widely distributed and extensive compared to RBs with the largest class of proteins involved in energy production. In RBs, the majority of phosphoproteins are involved in protein synthesis and folding. In this model, proteins involved in metabolism and virulence are phosphorylated to prime EBs for infection and energy production. Following entry into the host cell, reversible phosphorylation rapidly reorganizes the phosphoproteome to prepare for EB to RB differentiation. During RB development, proteins are further modulated to optimize metabolism, protein synthesis/folding, and other unidentified functions for growth and replication. Upon exposure to persistence inducing stimuli, *Chlamydia* enter a persistent state (PS) associated with a global transcriptional response that may ultimately lead to altered proteomic profiles, which in turn impact the phosphoproteomic landscape. Upon removal of the persistence inducing stimuli, the phosphoproteome would shift back to the RB phosphoproteome, which likely varies throughout development and converges on the EB phosphoproteome during RB to EB differentiation. Bars represent the relative number of proteins in EB (orange) and RB (purple) phosphoproteomes within each functional category. Solid arrows indicate the flow of phosphorylation and thickness is proportional to the abundance of phosphoproteins.

Global protein phosphorylation in prokaryotes has more recently been appreciated for its role in regulating important biological processes through reversible modification of protein function by protein kinases and phosphatases (Mijakovic and Macek, 2012). Protein phosphorylation controls a broad range of processes such as development, virulence, and adaptive responses through dynamic control of enzyme activity, protein localization, signal transduction, and protein oligomerization (Pereira et al., 2011; Grangeasse et al., 2012). The reduced genomes of *Chlamydia* encode a limited, but we hypothesize important, arsenal of phosphorylation-related proteins. This review examines the current knowledge of protein phosphorylation in chlamydial physiology and development, summarized in Figure 1, and highlights future avenues for exploration.

## Phosphoproteome

Phosphoproteomic analysis of numerous bacteria has established that while less prevalent than protein phosphorylation in eukaryotes, which can phosphorylate greater than 50% of their proteome, protein phosphorylation is an integral feature of bacterial physiology and pathogenesis (Jers et al., 2008; Olsen et al., 2010). Phosphoproteomic analysis has been performed with the EB and RB forms of *Chlamydia caviae*, a Guinea pig pathogen with 80% of its genes having homologs in the human pathogens *Chlamydia trachomatis* and *Chlamydia pneumoniae* (Read et al., 2003). The *C. caviae* phosphoproteome contains at least 42 proteins (4% of the proteome) of which 41 have homologs in all sequenced *Chlamydia* (Fisher et al., 2015). Consistent with the promiscuous nature of Hank's type kinases (Pereira et al., 2011), the number of phosphorylated proteins exceeds the number of known functional chlamydial protein kinases (two). The phosphoproteins are differentially allocated between EB (74%) and RB (19%) forms with only three proteins shared between the two developmental forms. The EB phosphoproteome is enriched for proteins involved in central and secondary metabolism along with hypothetical and virulence proteins. In contrast, the RB phosphoproteome is primarily associated with protein synthesis and folding.

Among the phosphoproteins that were identified in EBs, the largest class consisted of proteins involved in energy production (23%). While EBs are metabolically inactive, proteome studies and axenic culturing conditions indicate that EBs contain, and can likely activate, the majority of enzymes involved in central and secondary metabolism suggesting that they are primed for metabolism upon entry into the host cell (Omsland et al., 2012). The majority of EB-phosphorylated metabolic proteins were not phosphorylated in RBs supporting the hypothesis that phosphorylation acts to rapidly modulate the activity of metabolic enzymes alleviating the need for transcriptional and translational machinery during immediate-early infection. In addition, the T3SS structural proteins CdsN and CdsD (phosphorylated *in vitro*, Johnson and Mahony, 2007) were phosphorylated in EBs, but not RBs. One hypothesis is that the T3SS may be held in an off state until contact is made with the host cell, in part, by phosphorylation.

The published chlamydial phosphoproteome is likely underrepresented as phosphoproteomes are dynamic and change over time and under different conditions. For example, we speculate that the phosphoproteome would vary between early, middle, and late stage growth and during persistence to fulfill the physiological needs of each developmental stage. While the asynchronous nature of the RB to EB transition, sensitivity of detection, and lability of phospho-modifications represent challenges for phosphoproteome mapping, newer mass spectrometry methods overcome many of these limitations and could be applied to multiple chlamydial species to obtain temporal phosphoproteome maps capturing phospho-site data (limited phospho-site information was obtained from the *C. caviae* study). To date, the phosphoproteomes of other obligate intracellular pathogens have not been examined. *Coxiella*, which encode multiple protein kinases and phosphatases (Table 1) and undergo developmental alterations, would appear to be the ideal candidate for phosphoproteome analysis (Minnick and Raghavan, 2012).

**Table 1:** **Summary of phosphoproteins in pathogenic obligate intracellular bacteria**.

	***C. trachomatis* D/UW-3/CX^a^**	***Coxiella burnetii* RSA 493**	***Rickettsia rickettsii* str. Hlp#2**	***Anaplasma phagocytophilum* str. HZ**	***Ehrlichia chaffeensis* str. Arkansas**
Protein kinase	CT_145 CT_301 CT_673	CBU_0175 CBU_1168^b^ CBU_1379^b^	NA	NA	NA
Protein phosphatase	CT_259	CBU_0488 CBUA0032^c^	NA	NA	NA
Rsb—partner switching mechanism	CT_424 CT_549 CT_588 CT_589 CT_765	NA^d^	NA	NA	NA
Histidine kinase	CT_467	CBU_0789 CBU_1761 CBU_2005^e^	RPK_RS00325 RPK_RS01565 RPK_RS02040 RPK_RS02045 RPK_RS04660	APH_RS00610 APH_RS02470^f^ APH_RS03745	ECH_RS01200 ECH_RS03125^f^ ECH_RS03640
Response regulator	CT_468 CT_630	CBU_0712 CBU_0760	RPK_RS00525 RPK_RS01630 RPK_RS02880	APH_RS02370 APH_RS04365	ECH_RS03205 ECH_RS04140
Arginine kinase	CT_675	NA	NA	NA	NA

a *Locus tags of predicted genes in each category are based on genome annotation. Chlamydia trachomatis D/UW-3/CX, NC_000117.1; Coxiella burnetii RSA 493, NC_002971.3 (chromosome) and NC_004704.1 (plasmid); Rickettsia rickettsii str. Hlp#2, NC_016915.1; Anaplasma phagocytophilum str. HZ, NC_007797.1; and Ehrlichia chaffeensis str. Arkansas, NC_007799.1*.

b *Frameshifted ORF*.

c *Plasmid-encoded*.

d *Not Annotated*.

e *Histidine Kinase-like*.

f *Hybrid Sensor Histidine Kinase/Response Regulator*.

## Protein kinases and phosphatases

First discovered in eukaryotes, reversible protein phosphorylation on Ser/Thr/Tyr residues has gained a wider appreciation for its diverse role in regulating bacterial physiology (Deutscher and Saier, 2005). Ser/Thr phosphorylation and Tyr phosphorylation are typically mediated by Hank's type kinases and bacterial tyrosine kinases (BY), respectively (Cousin et al., 2013). These enzymes use ATP as the phospho-donor and are known to be promiscuous regarding their protein substrate preference (Pereira et al., 2011). P-Ser/Thr/Tyr residues, unlike P-Asp and P-His residues found in two-component signaling systems, form more stable ester linkages requiring dedicated protein phosphatases working in conjunction with their cognate kinase (Klumpp and Krieglstein, 2002). Protein phosphatases are divided into two superfamilies: protein Ser/Thr phosphatases (PSPs), which are subdivided into phosphoprotein phosphatases (PPP) and protein phosphatases metal-dependent (PPM), and protein tyrosine phosphatases (PTPs) (Shi, 2009). BY kinases and PTPs are strongly associated with virulence (Whitmore and Lamont, 2012). In addition, an arginine kinase, McsB, plays a role in targeting proteins for degradation by the Clp protease system in some bacteria (Trentini et al., 2016).

All sequenced *Chlamydia* encode two validated Hank's type kinases, Pkn1 (cytoplasmic) and PknD (inner membrane), and a putative pseudokinase, Pkn5 (T3SS effector) (Verma and Maurelli, 2003; Johnson and Mahony, 2007), along with a putative arginine kinase, McsB. Pkn1 and PknD from *C. trachomatis* have been shown to autophosphorylate on Ser/Thr residues and to interact with each other, suggesting cross-regulation (Verma and Maurelli, 2003). *In vitro* approaches identified the inclusion membrane protein IncG as a Pkn1 substrate for *C. trachomatis* (Verma and Maurelli, 2003) and the T3SS structural protein CdsD as a PknD substrate for *C. pneumoniae* (Johnson and Mahony, 2007). While Pkn1 can phosphorylate IncG *in vitro*, IncG is likely phosphorylated by a host kinase *in vivo*, similar to IncA (Rockey et al., 1997; Scidmore and Hackstadt, 2001). Pkn1, PknD, and Pkn5 are transcribed throughout development and the proteins have been detected in the RB form using mass spectrometry or Western blot (Belland et al., 2003b; Verma and Maurelli, 2003; Albrecht et al., 2010; Saka et al., 2011; Skipp et al., 2016). Interestingly, two forms of PknD have been detected indicating that it may function in its full length membrane-bound form and as a truncated, cytoplasmic form containing the kinase domain (Verma and Maurelli, 2003).

While Pkn1 and PknD from *C. trachomatis* were found to phosphorylate Ser/Thr residues, but not Tyr (Verma and Maurelli, 2003), PknD from *C. pneumoniae* was shown to autophosphorylate on Thr/Tyr residues and to phosphorylate Ser/Tyr residues on CdsD (Johnson and Mahony, 2007). Tyr-phosphorylation by a Hank's type kinase is unusual and suggests a relaxed substrate specificity for the *C. pneumoniae* PknD. Sequence analysis and *in vitro* studies suggest that Pkn5 is a pseudokinase as it lacks conserved catalytic residues (Verma and Maurelli, 2003). Pkn5 is encoded in an operon containing T3SS genes and has been shown to serve as a T3SS substrate in a *Salmonella* surrogate T3SS assay (Ho and Starnbach, 2005). In addition, Pkn5 from *C. pneumoniae* localizes to the inclusion membrane (Herrmann et al., 2006) further supporting its role as an effector protein.

In contrast to most other bacteria, the protein kinases, phosphatases, and substrates do not appear to be encoded within operons in *Chlamydia* complicating the mapping of the phosphoprotein network. We recently characterized a protein phosphatase from *C. trachomatis*, Cpp1, which we hypothesize to partner with Pkn1 and PknD completing a reversible phosphoprotein network (Claywell and Fisher, 2016). All sequenced *Chlamydia* spp. encode Cpp1, which is a member of the PPM family of phosphatases. Additional PPM proteins in *Chlamydia* include RsbU and CT_589, a paralog of RsbU, which are assigned to the partner switching mechanism (Hua et al., 2006). Cpp1 is a member of the protein phosphatase type 2C (PP2C) subfamily of PPMs and is able to dephosphorylate P-Ser, P-Thr, and P-Tyr. Dephosphorylation of P-Tyr is not a common feature of PP2Cs. The broad substrate preference of Cpp1 in conjunction with the unique ability of the *C. pneumoniae* PknD to phosphorylate Tyr residues supports the presence of P-Tyr in *Chlamydia* despite the absence of PTP and BY kinases, which are present in the larger genomes of chlamydial ancestors (Collingro et al., 2011). Similar broadening of substrate specificity leading to loss of genes encoding proteins with narrow substrate preferences has also been seen for the chlamydial nucleotide transporters (Fisher et al., 2013). Studies should be pursued to address whether tyrosine phosphorylation is present in other *Chlamydia* or if it is restricted to *C. pneumoniae*.

Numerous studies support the importance of protein phosphorylation to chlamydial growth and virulence. A *Chlamydia psittaci* strain containing a radical mutation on a conserved Ser residue of Pkn5, which serves as a potential phosphorylation and/or binding site for host proteins, is attenuated for virulence in a mouse pneumonia model (Miyairi et al., 2011), and chemically induced *C. trachomatis* mutants possessing small plaque morphologies carry missense mutations mapping back to the kinases and Cpp1 (Kokes et al., 2015). In addition, the absence of nonsense mutations for PknD/Pkn1/Cpp1 indicates that these enzymes may be essential for chlamydial growth (Kokes et al., 2015). Finally, inhibition of PknD (Johnson et al., 2009) significantly reduces growth of *C. pneumoniae* further suggesting that protein phosphorylation is integral to chlamydial physiology and that these enzymes could be therapeutic targets.

## Regulation of transcription through the partner switching mechanism and two-component signaling systems

Adaptive responses are frequently mediated by differential gene expression in response to external stimuli. Bacteria often perform these tasks using two-component signaling systems, which are found in *Chlamydia* and other obligate intracellular bacteria (Table 1), and less frequently via partner switching mechanisms (PSMs). *Chlamydia* encode a regulator of sigma B (Rsb)-type PSM, which has been extensively studied in *Bacillus* (Wise and Price, 1995). This regulatory mechanism controls the availability of sigma factors in *Bacillus* by a series of protein-protein interactions that are themselves regulated by phosphorylation (Hecker and Völker, 2001). In the *Bacillus* PSM module, the anti-sigma factor RsbW phosphorylates the anti-anti-sigma factor RsbV, freeing itself to sequester the target sigma factor. In the presence of a signal, the sensor phosphatase RsbU dephosphorylates RsbV, leading to binding of RsbV to RsbW and release of the sigma factor. The free ratio of the sigma factor is dependent upon levels of PSM proteins and the activity of the sensor phosphatase.

In *Chlamydia*, the PSM includes one validated (RsbU) and one putative sensor phosphatase (CT_589 which lacks critical PP2C residues), two anti-anti-sigma factors, RsbV1 and RsbV2, and an anti-sigma factor, RsbW (Douglas and Hatch, 2000; Hua et al., 2006). The chlamydial PSM appears to modulate the availability of σ^66^, the major sigma factor responsible for transcription of housekeeping genes (Thompson et al., 2015). When RsbV1 was overexpressed in *C. trachomatis*, transcription of σ^66^ regulated genes was upregulated and the cells exhibited increased growth, whereas the inverse occurred when RsbW was overexpressed or when RsbV1 was inactivated. Consistent with the classical model, RsbW and RsbU are able to govern the phosphorylation status of RsbV1. In contrast, RsbV2, which is phosphorylated by RsbW (at reduced rates compared to RsbV1), could not be dephosphorylated by RsbU. The current working PSM model suggests that it serves as a molecular throttle on metabolic activity and consequently growth rate. PSM mutant strains are currently being put through normal and “stress” conditions to further test the PSM model. Ligands controlling the sensor phosphatases, the roles of CT_589 and RsbV2, and whether other targets for RsbW and RsbV2 exist remain to be explored.

Two-component signaling systems (TCSS) enable adaptive responses to changing environmental conditions (Mitrophanov and Groisman, 2008). Obligate intracellular bacterial pathogens, unlike most free-living bacteria, are reliant on the host for development and appear to encode a limited number of TCSSs. In the classical form, TCSSs consist of the integral membrane sensor histidine kinase (HK) and a cytoplasmic response regulator (RR). Various signals (pH, temperature, osmolarity, etc.) lead to autophosphorylation of the HK on a conserved histidine residue and subsequent phospho-transfer to the receiver domain of a cognate RR on an invariant aspartate residue. Classical RRs oligomerize and bind to DNA through their output domains leading to alterations in transcription.

*Chlamydia* spp. possess a single complete TCSS along with an orphaned RR (Koo and Stephens, 2003; Koo et al., 2006). The chlamydial HK and cognate RR, CtcB and CtcC, are homologous to NtrB/AtoS and NtrC/AtoC, respectively, and are predicted to modulate the expression of σ^54^ regulated genes. CtcC-type activators promote transcription by binding DNA at enhancers, forming oligomers, and inducing ATPase activity that converts closed complexes to open complexes (Tucker and Sallai, 2007). Interestingly, CtcC lacks the helix-turn-helix domain responsible for DNA binding among σ^54^ activators (Koo and Stephens, 2003). Dimeric and tetrameric forms of CtcC were detected in the absence of DNA, suggesting enhancer binding is not necessary for σ^54^ RNA polymerase holoenzyme activation. While the physiological role of CtcB/CtcC is not yet known, this system is developmentally regulated as transcripts and protein levels are present during late development and are speculated to play a role in RB to EB differentiation. In addition to CtcB and CtcC, *Chlamydia* also encode ChxR, a response regulator that is homologous to the OmpR subfamily, but is atypical in that the receiver domain lacks the invariant Asp residue (Koo et al., 2006). ChxR appears capable of dimerizing and binding DNA in the absence of phosphorylation, consistent with the absence of both the conserved aspartate and a cognate HK (Hickey et al., 2011). Similar to CtcB/CtcC, a defined role for ChxR in chlamydial physiology is lacking.

## Chlamydial proteins phosphorylated by host kinases

*Chlamydia* can deliver proteins into both the inclusion lumen and host cytoplasm using the T3SS and a type 2 secretion system (Hsia et al., 1997; Nguyen and Valdivia, 2012). At least four of these proteins, translocated actin-recruiting protein (TarP), translocated early phosphoprotein (TepP), and inclusion membrane proteins A and G (IncA/IncG), are known to be phosphorylated by host kinases (Rockey et al., 1997; Scidmore and Hackstadt, 2001; Clifton et al., 2005; Chen et al., 2014). While phosphorylation of TarP and TepP likely play roles in chlamydial entry and inclusion formation, it is less clear what role phosphorylation plays in the functions of IncA/IncG. Of note, sequence diversity for both TarP, including absence of the tyrosine phosphorylation motif in non-*C. trachomatis* species, and IncA indicate that phosphorylation of the homologs may not occur in all chlamydial species (Bannantine et al., 1998; Delevoye et al., 2004; Clifton et al., 2005). Two recent studies mapping the Inc protein interactome and the inclusion proteome identified multiple host kinases and phosphatases in association with the inclusion proteins or inclusion making it highly likely that other chlamydial proteins are reversibly phosphorylated by host enzymes (Aeberhard et al., 2015; Mirrashidi et al., 2015). How phosphorylation of these proteins contributes to the dynamic interplay between the bacterium and host will be an exciting topic for future research.

## Concluding remarks

Protein phosphorylation is a widely employed post-translational modification that mediates important processes in bacteria including cell signaling, enzyme activity, and protein-protein interactions contributing to bacterial growth and virulence. *Chlamydia* and other obligate intracellular bacterial pathogens appear to encode a limited number of two-component signaling systems compared to most free-living bacteria (Table 1), which may reflect their restricted environmental niche. In addition, only *Chlamydia* and *Coxiella* seem capable of Ser/Thr/Tyr protein phosphorylation, and *Chlamydia* is the lone species possessing a PSM. While limited in scope, the maintenance of these phosphorylation pathways by minimal genome organisms suggests that they are vital for bacterial survival and pathogenesis, and chlamydial mutagenesis and inhibitor studies are supportive of an integral role for phosphorylation in development and growth.

More research is needed to elucidate the physiological role of protein phosphorylation in *Chlamydia* and other obligate intracellular pathogens. Despite the validation of chlamydial protein kinases, phosphatases, TCSSs, and the sole PSM, their roles *in vivo* remain unclear. In addition, the Ser/Thr/Tyr phosphoprotein network and an understanding of the functional consequences of substrate phosphorylation are far from complete. Fortunately, the burgeoning number of genetic methods now available for use with *Chlamydia* (Bastidas and Valdivia, 2016) along with rapidly improving methods for studying phosphoproteomes and transcriptomes should empower researchers to address these significant gaps in our knowledge of chlamydial physiology and pathogenesis.

## Author contributions

DF conceived of the topic and helped with writing and editing of the manuscript. JC and LM contributed ideas, wrote the manuscript, and helped with editing.

## Funding

Research reported in this publication was supported by the NIAID of the National Institutes of Health under Award Number 1R15AI109566-01A1 to DF. The content is solely the responsibility of the authors and does not necessarily represent the official views of the National Institutes of Health.

### Conflict of interest statement

The authors declare that the research was conducted in the absence of any commercial or financial relationships that could be construed as a potential conflict of interest.
